# GibbsST: a Gibbs sampling method for motif discovery with enhanced resistance to local optima

**DOI:** 10.1186/1471-2105-7-486

**Published:** 2006-11-04

**Authors:** Kazuhito Shida

**Affiliations:** 1 TUBERO(Tohoku University Biomedical Engineering Research Organization) 980-8575, Sendai, Japan

## Abstract

**Background:**

Computational discovery of transcription factor binding sites (TFBS) is a challenging but important problem of bioinformatics. In this study, improvement of a Gibbs sampling based technique for TFBS discovery is attempted through an approach that is widely known, but which has never been investigated before: reduction of the effect of local optima.

**Results:**

To alleviate the vulnerability of Gibbs sampling to local optima trapping, we propose to combine a thermodynamic method, called simulated tempering, with Gibbs sampling. The resultant algorithm, GibbsST, is then validated using synthetic data and actual promoter sequences extracted from *Saccharomyces cerevisiae*. It is noteworthy that the marked improvement of the efficiency presented in this paper is attributable solely to the improvement of the search method.

**Conclusion:**

Simulated tempering is a powerful solution for local optima problems found in pattern discovery. Extended application of simulated tempering for various bioinformatic problems is promising as a robust solution against local optima problems.

## Background

One of the most important and challenging problems in post-genomic stage of bioinformatics is the automated TFBS discovery [1]; computational identification of potential binding sites in upstream region of genes, which is a necessary step to understand the regulatory network within the living cell. These binding sites can be identified as over-represented and over-preserved short segments in the upstream sequences by means of a local alignment. In this problem, local alignments are usually assumed to be gapless and can be represented by a number of starting points in the input sequences. Apparently, this is a multivariate optimization problem.

Optimization problems with large numbers of parameters are generally prone to the problem of local optima, and discovery of TFBS (and any pattern with biological importance) is no exception. In particular, one of the most promising types of stochastic pattern discovery methods in terms of its flexibility and wide range of application, generically called Gibbs sampling [2], is known to be rather strongly affected by the local optima problem [3]. In theory, the stochastic nature of Gibbs sampling is presumed to prevent it from becoming trapped completely in a local optimum. In practice, because of the strong disturbance from local optima, Gibbs sampling requires initial values that are set sufficiently close to the global optimum for reliable convergence. Practical but inefficient solutions to this problem are performing numerous independent Gibbs sampling runs with different initial conditions, or merely resorting to extremely long runs, hoping that the global optimum will be attained. In short, Gibbs sampling has ample room for improvement as a search method in the solution space.

In pattern discovery and bioinformatics in general, improvement of search methods in the solution space has been neither systematic nor satisfactory. The method most frequently tried is the simulated annealing(SA) [4-6]. Frith et al. [7] tested a few different annealing procedures, but these resulted in a performance gain of only a few percentage points. Improvement of the selection of initial parameters is of course possible, namely, by a heuristic approach [8]. However, it is unclear how helpful such heuristic guidance would be when patterns have much larger variations.

In general, there has been a real disparity between the lack of interest in improving the search methods and the strong interest in creating new models for TFBS discovery. Moreover, the active introduction of new ideas into this field is making the disparity even stronger, because many of the new ideas are related to increasing the number of parameters. For example, automated phylogenetic footprinting [9,10] is a promising way to improve detection performance, but it involves more parameters than the conventional methods because it takes the phylogenetic mutation history and the parameters to model that history into account (even when the phylogenetic parameters are not optimized to avoid over-fitting, the situation is basically the same). There have been many other recent proposals involving an increased number of parameters in the model, including the improvement of the background model by a higher-order Markov model [11], the simultaneous optimization of multiple models [12], the introduction of site-site dependence (co-evolution) into the mutational model of TFBS [13]. There is no guarantee that improvement of sensitivity and specificity by improved model and score function always make their score-landscape more smooth. Many benefits of sophisticated models can be easily vanished due to the "dimensional curse" of the increased number of parameters, unless proper consideration is made for the search method as well.

**Figure 1:**
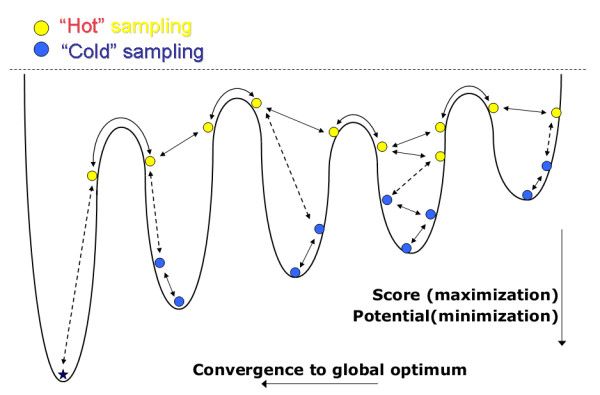
**Principle of simulated tempering**. This figure illustrates a thermodynamic depiction of an optimization problem, the local optima difficulty in iterative optimization procedures (shown by solid arrows), and how it can be alleviated by temperature level transitions (shown by dashed arrows).

In this paper, we demonstrate that simulated tempering (ST) [14], which is one of many proposals from the field of thermodynamics for the systematic avoidance of local optima in multivariate optimization problems, is quite useful for reducing the vulnerability of Gibbs sampling to local optima. The application of ST to a genetics problem has already been reported [15]. SA and potential deformation [16,17], which has already succeeded in other problems of bioinformatics, are also rooted in the field of thermodynamics. ST and SA employ a new parameter called "*temperature*" *T*, the introduction of which into a local-alignment problem has already been reported [18]. The novelty of ST is that it attempts to adjust the value of *T *adaptively to the current score of alignments. By changing *T*, ST adopts continuously changing search methods ranging from a fast deterministic-like search to a random-like search, reducing the possibility of being trapped in local optima. This principal is schematically shown in Fig. 1. In the present work, we implemented and tested an ST-enhanced Gibbs sampling algorithm for TFBS discovery, which we call *GibbsST*. The validation of our algorithm is also presented on synthetic test data and promoter sequences of *Saccharomyces cerevisiae*.

## Results

### Gibbs sampling with temperature

In this section, we introduce a temperature, *T*, into the "classic" Gibbs sampling algorithm proposed by Lawrence et al. [2] The details of the algorithm (row selection order, pseudocount, etc.) will be introduced later along with the implementation of our algorithm. For simplicity, it is assumed that all *N *of input sequences have exactly one occurrence (the OOPS-model) of the pattern, which is always *W*_*m *_bp long, and negative strands are not considered.

The algorithm holds a current local alignment, *A*, and a current PWM (Position Weight Matrix), *q*_*i,j*_, which are iteratively updated as a Markov chain until the convergence to a pattern. The alignment *A *is represented by the starting points of aligned segments, *x*_*k*_, which form a gapless sequence block. The first half of an iterative step is the re-calculation of elements of the current PWM according to the current alignment, excluding the *k*-th row. Then in the second half of a step, the *k*-th row of the current alignment is updated by sampling a new value of *x*_*k *_according to weights derived from *q*_*i,j*_. Let *l*(1), *l*(2), ... denote the entire sequence of the row to be updated. We set the probability of the new starting point being *x *proportional to

(QxPx)β,β=1/T,     (1)
 MathType@MTEF@5@5@+=feaafiart1ev1aaatCvAUfKttLearuWrP9MDH5MBPbIqV92AaeXatLxBI9gBaebbnrfifHhDYfgasaacH8akY=wiFfYdH8Gipec8Eeeu0xXdbba9frFj0=OqFfea0dXdd9vqai=hGuQ8kuc9pgc9s8qqaq=dirpe0xb9q8qiLsFr0=vr0=vr0dc8meaabaqaciaacaGaaeqabaqabeGadaaakeaacqGGOaakdaWcaaqaaiabdgfarnaaBaaaleaacqWG4baEaeqaaaGcbaGaemiuaa1aaSbaaSqaaiabdIha4bqabaaaaOGaeiykaKYaaWbaaSqabeaaiiGacqWFYoGyaaGccqGGSaalcqWFYoGycqGH9aqpcqaIXaqmcqGGVaWlcqWGubavcqGGSaalcaWLjaGaaCzcamaabmaabaGaeGymaedacaGLOaGaayzkaaaaaa@4125@

where Qx=∏i=0Wm−1ql(x+i),i
MathType@MTEF@5@5@+=feaafiart1ev1aaatCvAUfKttLearuWrP9MDH5MBPbIqV92AaeXatLxBI9gBaebbnrfifHhDYfgasaacH8akY=wiFfYdH8Gipec8Eeeu0xXdbba9frFj0=OqFfea0dXdd9vqai=hGuQ8kuc9pgc9s8qqaq=dirpe0xb9q8qiLsFr0=vr0=vr0dc8meaabaqaciaacaGaaeqabaqabeGadaaakeaacqWGrbqudaWgaaWcbaGaemiEaGhabeaakiabg2da9maaradabaGaemyCae3aaSbaaSqaaiabdYgaSjabcIcaOiabdIha4jabgUcaRiabdMgaPjabcMcaPiabcYcaSiabdMgaPbqabaaabaGaemyAaKMaeyypa0JaeGimaadabaGaem4vaC1aaSbaaWqaaiabd2gaTbqabaWccqGHsislcqaIXaqma0Gaey4dIunaaaa@450B@ is the likelihood that the *x*-th substring (*x *~ *x *- 1 + *W*_*m *_-th letters) of the *k*-th input sequence comes from the probabilistic model represented by the current PWM, and Px=∏i=0Wm−1pl(x+i)
MathType@MTEF@5@5@+=feaafiart1ev1aaatCvAUfKttLearuWrP9MDH5MBPbIqV92AaeXatLxBI9gBaebbnrfifHhDYfgasaacH8akY=wiFfYdH8Gipec8Eeeu0xXdbba9frFj0=OqFfea0dXdd9vqai=hGuQ8kuc9pgc9s8qqaq=dirpe0xb9q8qiLsFr0=vr0=vr0dc8meaabaqaciaacaGaaeqabaqabeGadaaakeaacqWGqbaudaWgaaWcbaGaemiEaGhabeaakiabg2da9maaradabaGaemiCaa3aaSbaaSqaaiabdYgaSjabcIcaOiabdIha4jabgUcaRiabdMgaPjabcMcaPaqabaaabaGaemyAaKMaeyypa0JaeGimaadabaGaem4vaC1aaSbaaWqaaiabd2gaTbqabaWccqGHsislcqaIXaqma0Gaey4dIunaaaa@42CC@ is the likelihood that the same subsequence comes from a totally random sequence of the base composition observed for the entire input, *p*_0,1,2,3 _(that is, *p*_*G,A,C,T*_). The *T *is a positive value which is the "temperature" of the system. Note that the computational complexity of the single step of the optimization is not changed by introducing the temperature.

It is easy to see that the above introduced iteration step maximizes ∏i=0Wm−1(ql(x+i),i/pl(x+i))β
MathType@MTEF@5@5@+=feaafiart1ev1aaatCvAUfKttLearuWrP9MDH5MBPbIqV92AaeXatLxBI9gBaebbnrfifHhDYfgasaacH8akY=wiFfYdH8Gipec8Eeeu0xXdbba9frFj0=OqFfea0dXdd9vqai=hGuQ8kuc9pgc9s8qqaq=dirpe0xb9q8qiLsFr0=vr0=vr0dc8meaabaqaciaacaGaaeqabaqabeGadaaakeaadaqeWaqaaiabcIcaOiabdghaXnaaBaaaleaacqWGSbaBcqGGOaakcqWG4baEcqGHRaWkcqWGPbqAcqGGPaqkcqGGSaalcqWGPbqAaeqaaOGaei4la8IaemiCaa3aaSbaaSqaaiabdYgaSjabcIcaOiabdIha4jabgUcaRiabdMgaPjabcMcaPaqabaGccqGGPaqkdaahaaWcbeqaaGGaciab=j7aIbaaaeaacqWGPbqAcqGH9aqpcqaIWaamaeaacqWGxbWvdaWgaaadbaGaemyBa0gabeaaliabgkHiTiabigdaXaqdcqGHpis1aaaa@4E0A@, unless *T *is extremely large. Since *k *circulates all *N *of input sequences, this is a maximization of *β *∑ ∑ *q*_*i,j *_log(*q*_*i,j*_/*p*_*i*_) after all. Hence, the Gibbs sampling introduced here has the relative entropy of the pattern PWM against the background model as its goal-function (or score) to be maximized, and so does our algorithm.

However, following the convention of statistical physics, we refer to TFBS discovery as a minimization of the *potential U*, which is currently ( – relative entropy). Because we are not proposing a new definition of *U*, we do not evaluate the sensitivity and specificity of our new algorithm. In principle, the sensitivity and specificity must be independent from the search method in the limit of large step number.

When *T *= *β *= 1, it is reduced to the classic Gibbs sampling without the idea of temperature. In this case, there always is a finite probability of selection of non-optimal *x*, which gives rise to the escape from the local minima. However, the magnitude of the escape probability may not be sufficient for deep local minima, because the probability is ultimately limited by the pseudocount.

The temperature strongly affects the behavior of the optimization algorithm. It is easy to see that when *T *is large enough, the *x *selection is almost random (*T *→ ∞ means that the probabilities of all *x *are 1), and the algorithm is very inefficient despite the high immunity to the local minima problem. When *T *→ 0, on the other hand, a very quick convergence to local minima *only *results, because the movement in the solution space is a "steepest-descent" movement. In simulated annealing, the temperature is initially set to an ideally large value, *T*_*h*_, where essentially no barrier exists in the potential landscape, and then slowly lowered. There is a theoretical guarantee that SA converges to the global minimum when the temperature decreases slowly enough [19]. However, it is frequently unrealistic to follow the theory because of the large number of iterations required for annealing.

### Temperature scheduling

Simulated tempering is an accelerated version of simulated annealing and has two main features. First, the temperature of the system is continuously adjusted during the optimization process and may be increased as well as decreased. Second, the adjustment of temperature is performed without detailed analysis of the potential landscape. Temperature control is performed by introducing the second Markov chain (i.e. a random walk along the temperature axis) that is coupled with *U*.

In ST, the temperature of the system takes one of the *N*_*T *_temperature levels, *T*_0 _<*T*_1 _<*T*_2 _... <TNT−1
 MathType@MTEF@5@5@+=feaafiart1ev1aaatCvAUfKttLearuWrP9MDH5MBPbIqV92AaeXatLxBI9gBaebbnrfifHhDYfgasaacH8akY=wiFfYdH8Gipec8Eeeu0xXdbba9frFj0=OqFfea0dXdd9vqai=hGuQ8kuc9pgc9s8qqaq=dirpe0xb9q8qiLsFr0=vr0=vr0dc8meaabaqaciaacaGaaeqabaqabeGadaaakeaacqWGubavdaWgaaWcbaGaemOta40aaSbaaWqaaiabdsfaubqabaWccqGHsislcqaIXaqmaeqaaaaa@3274@ (usually, it is required that TNT−1
 MathType@MTEF@5@5@+=feaafiart1ev1aaatCvAUfKttLearuWrP9MDH5MBPbIqV92AaeXatLxBI9gBaebbnrfifHhDYfgasaacH8akY=wiFfYdH8Gipec8Eeeu0xXdbba9frFj0=OqFfea0dXdd9vqai=hGuQ8kuc9pgc9s8qqaq=dirpe0xb9q8qiLsFr0=vr0=vr0dc8meaabaqaciaacaGaaeqabaqabeGadaaakeaacqWGubavdaWgaaWcbaGaemOta40aaSbaaWqaaiabdsfaubqabaWccqGHsislcqaIXaqmaeqaaaaa@3274@ ~ *T*_*h*_). During the optimization, the temperature is updated accordingly to the transition rates, *R*, given by a Metropolis-Hastings-like formula:

*R*(*T*_*i *_→ *T*_*i *+ 1_) ∝ 1/(1 + *S*_+_)     (2)

*R*(*T*_*i *_→ *T*_*i *- 1_) ∝ *S*_-_/(1 + *S*_-_),     (3)

where *S*_± _is given by

Zi±1Ziexp⁡(−U/Ti)exp⁡(−U/Ti±1).     (4)
 MathType@MTEF@5@5@+=feaafiart1ev1aaatCvAUfKttLearuWrP9MDH5MBPbIqV92AaeXatLxBI9gBaebbnrfifHhDYfgasaacH8akY=wiFfYdH8Gipec8Eeeu0xXdbba9frFj0=OqFfea0dXdd9vqai=hGuQ8kuc9pgc9s8qqaq=dirpe0xb9q8qiLsFr0=vr0=vr0dc8meaabaqaciaacaGaaeqabaqabeGadaaakeaadaWcaaqaaiabdQfaAnaaBaaaleaacqWGPbqAcqGHXcqScqaIXaqmaeqaaaGcbaGaemOwaO1aaSbaaSqaaiabdMgaPbqabaaaaOWaaSaaaeaacyGGLbqzcqGG4baEcqGGWbaCcqGGOaakcqGHsislcqWGvbqvcqGGVaWlcqWGubavdaWgaaWcbaGaemyAaKgabeaakiabcMcaPaqaaiGbcwgaLjabcIha4jabcchaWjabcIcaOiabgkHiTiabdwfavjabc+caViabdsfaunaaBaaaleaacqWGPbqAcqGHXcqScqaIXaqmaeqaaOGaeiykaKcaaiabc6caUiaaxMaacaWLjaWaaeWaaeaacqaI0aanaiaawIcacaGLPaaaaaa@5427@

The *Z*_*i *_are a normalizing factor usually called the partition function of the system, defined as

Zi=∑exp⁡(−UTi).     (5)
 MathType@MTEF@5@5@+=feaafiart1ev1aaatCvAUfKttLearuWrP9MDH5MBPbIqV92AaeXatLxBI9gBaebbnrfifHhDYfgasaacH8akY=wiFfYdH8Gipec8Eeeu0xXdbba9frFj0=OqFfea0dXdd9vqai=hGuQ8kuc9pgc9s8qqaq=dirpe0xb9q8qiLsFr0=vr0=vr0dc8meaabaqaciaacaGaaeqabaqabeGadaaakeaacqWGAbGwdaWgaaWcbaGaemyAaKgabeaakiabg2da9maaqaeabaGagiyzauMaeiiEaGNaeiiCaaNaeiikaGIaeyOeI0YaaSaaaeaacqWGvbqvaeaacqWGubavdaWgaaWcbaGaemyAaKgabeaaaaGccqGGPaqkaSqabeqaniabggHiLdGccqGGUaGlcaWLjaGaaCzcamaabmaabaGaeGynaudacaGLOaGaayzkaaaaaa@421F@

How should the temperature levels be decided in ST? Unlike the case of simulated annealing, no conclusive theory or rule is known for the decision of algorithmic parameters of simulated tempering, except for the requirement of small temperature intervals. According to the equations above, the equilibrium distributions of *U *defined for neighboring values of *T*_*i *_must be overlapped to ensure finite transition rates between these temperature levels. This mainly requires small temperature intervals.

The temperature levels must be decided empirically, which leaves us a vast combination of *T*_*i *_to explore. However, considering the success of classic Gibbs sampling (and our preliminary test, whose data are not shown), we can safely assume that *T*_*h *_~ 1 for the current problem. Moreover, a good starting point has already been pointed out by Frith et al. [7]. In their paper, they introduced temperature in a manner similar to ours, and reported that a slight improvement of performance was observed only when they fixed the temperature to slightly lower than 1. So, in this paper, we planned to test only five different settings of temperature levels, called TLC1 to 5 (TLC stands for "Temperature Levels Combination"), as shown in Table 1. For example, TLC1 must be pretty close to the already reported condition of fixed *T*. Then, we extend the temperature range toward low temperature regime, retaining access to the high-temperature regime by increasing the temperature interval.

**Table 1:** Temperature settings for GibbsST: the six TLCs (Temperature Level Combinations) tested in this paper.

Name	Temperature levels
TLC 1	0.94, 0.95, 0.96, 0.97, 0.98
TLC 2	0.82, 0.86, 0.90, 0.94, 0.98
TLC 3	0.66, 0.74, 0.82, 0.90, 0.98
TLC 4	0.58, 0.68, 0.78, 0.88, 0.98
TLC 5	0.50, 0.62, 0.74, 0.86, 0.98

TLC 6	0.46, 0.58, 0.70, 0.82, 0.94

TLC6 was used only in "Comparison with fixed-*T *methods".

"Classic" mode: *T *is always 1

The point of this experimental design is to investigate the trade-off between small *T*_0 _and small temperature interval. Small *T*_0 _lowers |*T*| and accelerates convergence until the temperature interval becomes too large for a smooth transition between temperature levels. The third possibility, increasing the number of temperature levels, *N*_*T*_, will be briefly examined in the discussion.

### Test code

We implemented our new algorithm, called "*GibbsST*", into a C++ code. By default, the code randomly selects 50 local alignments as initial values and starts independent GibbsST optimization runs from them. The results from these multiple runs are merged (the alignment with the largest score for given number of steps is reported) upon output. It is unrealistic to expect the current version of GibbsST to reach global optima from the fewer number of initial values. Also, the merging of multiple runs reduces the scatter of the resultant convergence profile, which is useful for evaluating our algorithm.

### Test on synthetic data

In this section, our algorithm is tested on various synthetic test datasets. The performance of our algorithm is evaluated as a function of the temperature settings, and the optimal performance will be compared to that of classic Gibbs sampling. Such an empirical approach is crucially important for ST because there is no conclusive theory regarding the determination of temperature levels of ST. Basically, our model for synthetic TFBS is the one proposed in the "motif-challenge" problem [20], although the level of variation, controlled by the number of mutations added to the synthetic consensus sequence, *d*, is quite limited by our validation scheme (see Methods).

Since our current goal is to make our algorithm less prone to the local optima problem, it is highly desirable that the synthetic datasets are well-characterized in terms of their global optimum alignment. If the true global optimum in a dataset (and *W*_*m*_) is known, a performance coefficient of the current answer can be defined. In this paper, we use a performance coefficient based on the segment overlap between two alignments [20], defined as

∑i=1Nmax⁡(0,Wm−|xi−yi|)/∑i=1Nmin⁡(Wm+|xi−yi|,2Wm),     (6)
 MathType@MTEF@5@5@+=feaafiart1ev1aaatCvAUfKttLearuWrP9MDH5MBPbIqV92AaeXatLxBI9gBaebbnrfifHhDYfgasaacH8akY=wiFfYdH8Gipec8Eeeu0xXdbba9frFj0=OqFfea0dXdd9vqai=hGuQ8kuc9pgc9s8qqaq=dirpe0xb9q8qiLsFr0=vr0=vr0dc8meaabaqaciaacaGaaeqabaqabeGadaaakeaadaaeWbqaaiGbc2gaTjabcggaHjabcIha4jabcIcaOiabicdaWiabcYcaSiabdEfaxnaaBaaaleaacqWGTbqBaeqaaOGaeyOeI0YaaqWaaeaacqWG4baEdaWgaaWcbaGaemyAaKgabeaakiabgkHiTiabdMha5naaBaaaleaacqWGPbqAaeqaaaGccaGLhWUaayjcSdGaeiykaKcaleaacqWGPbqAcqGH9aqpcqaIXaqmaeaacqWGobGta0GaeyyeIuoakiabc+caVmaaqahabaGagiyBa0MaeiyAaKMaeiOBa4galeaacqWGPbqAcqGH9aqpcqaIXaqmaeaacqWGobGta0GaeyyeIuoakiabcIcaOiabdEfaxnaaBaaaleaacqWGTbqBaeqaaOGaey4kaSYaaqWaaeaacqWG4baEdaWgaaWcbaGaemyAaKgabeaakiabgkHiTiabdMha5naaBaaaleaacqWGPbqAaeqaaaGccaGLhWUaayjcSdGaeiilaWIaeGOmaiJaem4vaC1aaSbaaSqaaiabd2gaTbqabaGccqGGPaqkcqGGSaalcaWLjaGaaCzcamaabmaabaGaeGOnaydacaGLOaGaayzkaaaaaa@6D92@

where *y*_*i *_is the starting positions of the segments forming the true global optimum. This is a very effective way to isolate the features of the goal-function, the sensitivity and specificity (schematically, they are relevant to the vertical depth of basins of Fig. 1), from the efficiency of the search method itself (this is relevant to efficient movement along the horizontal axis of the same figure). A local optima resistant algorithm must show a rapid increase of the averaged performance coefficient, even from randomly given initial conditions.

With 7 different modes our discovery code was applied to the synthetic datasets generated under the conditions shown in Table 2 : TLC1 to 5, classic Gibbs (*T *= 1.0), and proposal of Frith et al. (*T *= 0.9) were compared. To evaluate the average performance over various inputs, 100 independent datasets were generated and analyzed for each condition. The other algorithmic parameters were the same for all combinations.

**Table 2:** Characteristics of synthetic datasets.

Condition	
*W*_*m *_= 8, *N *= 12, *W*_*b *_= 600	*d *= 1
*W*_*m *_= 12, *N *= 10, *W*_*b *_= 1000	*d *= 1
*W*_*m *_= 12, *N *= 10, *W*_*b *_= 1000	*d *= 2
*W*_*m *_= 16, *N *= 10, *W*_*b *_= 1000	*d *= 1
*W*_*m *_= 16, *N *= 10, *W*_*b *_= 1000	*d *= 2
*W*_*m *_= 12, *N *= 10, *W*_*b *_= 1000	*d *= 3 ^1^

The parameters used for dataset generation in the six synthetic conditions: *W*_*m*_, *N*, *W*_*b*_, and *d *denote the width of the pattern, the number of input sequences, the length of the background sequences, and the number of mutations in a pattern occurrence, respectively.

^1 ^This was used only in "Comparison with fixed-*T *methods".

**Figure 2:**
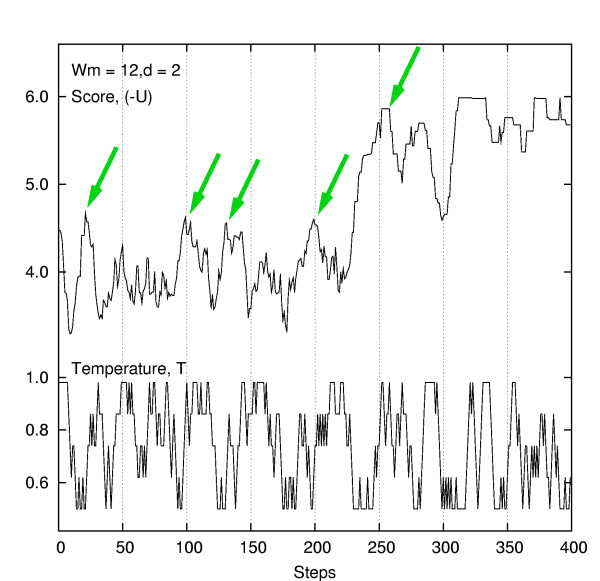
**Score, temperature and their interplay**. A typical time course of the value of score and *T *in the GibbsST iteration with TLC5.

Fig. 2 shows a typical time course of the value of score and *T *in a GibbsST iteration. This data obtained by TLC5 shows that the transition of temperature levels was smooth, suggesting that all TLCs tested were appropriate regarding their temperature intervals. Also, the plot illustrates how GibbsST solves the local optima problem; the optimization process encountered a series of local optima (shown by arrows), but GibbsST escaped from those local optima by increasing the temperature for a brief period, then resumed optimization exploiting the efficiency at lower temperature.

**Figure 3:**
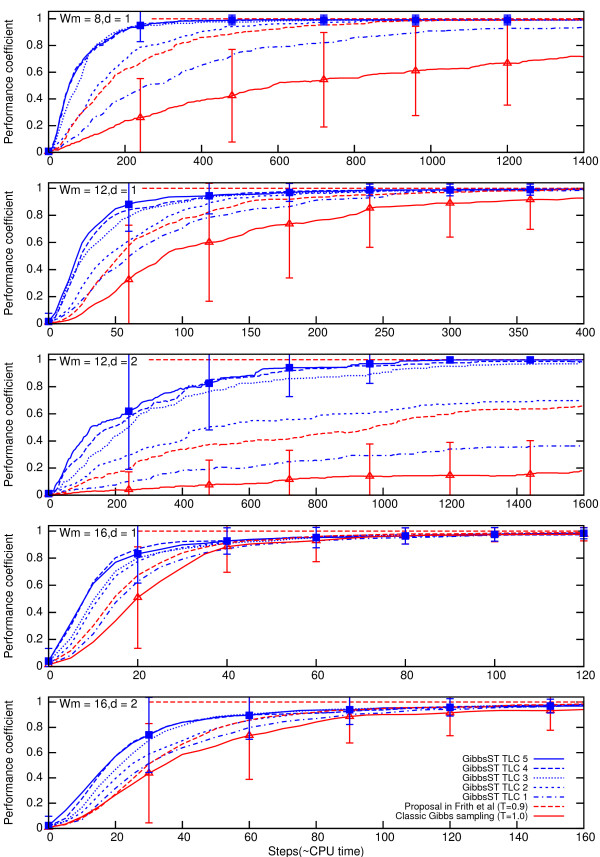
**Performance coefficient time course for synthetic datasets**. Average performance coefficient and its standard deviation (for classic Gibbs sampling and GibbsST with TLC5) for synthetic datasets.

Fig. 3 shows time course of the average performance coefficient (a plot of the performance coefficient versus the number of optimization steps) for various algorithm settings. Also, the standard deviation of the performance coefficient is shown as an error-bar for selected cases. In all pattern length and pattern variation level tested, the superiority of the GibbsST algorithm over the classic Gibbs sampling is vividly shown. The performance coefficient profile of GibbsST is always above that of classic Gibbs sampling. In many cases it smoothly converges to 1, which means the global optimum is reached. On the contrary, in some cases, classic Gibbs sampling shows extremely poor convergence to the global optimum because the randomly selected initial values were inappropriate for classic Gibbs sampling. There are statistically significant performance gaps between GibbsST (TLC5) and classic Gibbs sampling for all of the cases unless step number is too large (note that the standard error of performance coefficient is 1/100
 MathType@MTEF@5@5@+=feaafiart1ev1aaatCvAUfKttLearuWrP9MDH5MBPbIqV92AaeXatLxBI9gBaebbnrfifHhDYfgasaacH8akY=wiFfYdH8Gipec8Eeeu0xXdbba9frFj0=OqFfea0dXdd9vqai=hGuQ8kuc9pgc9s8qqaq=dirpe0xb9q8qiLsFr0=vr0=vr0dc8meaabaqaciaacaGaaeqabaqabeGadaaakeaadaGcaaqaaiabigdaXiabicdaWiabicdaWaWcbeaaaaa@2F93@ of the error-bars in the plot).

When *T *was fixed to 0.9, the performance was significantly improved in all cases tested. However, the extent of performance improvement was always smaller than that of GibbsST. It is interesting to note that *T *= 0.9 performed slightly poorer than TLC2 (the temperature was 0.9 at its central step).

We can conclude that GibbsST achieves a substantial improvement in performance over existing Gibbs sampling methods when the pattern length is small and the pattern-variation level is high. It is difficult to decide the optimal temperature setting because there is very little difference in performance among TLC3, 4 and 5, although TLC5 shows the best performance. For a further performance improvement, the use of lower *T*_0 _than that of TLC5 seems to deserve serious consideration.

### Comparison with fixed-*T *methods

**Figure 4:**
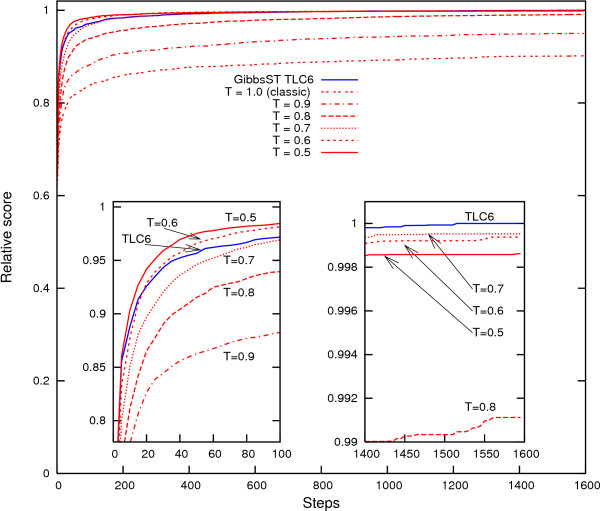
**GibbsST is superior to fixed-*T *methods for rough score landscape**. Time-course of average relative score of a dataset obtained by various temperature settings for a special dataset with rough score landscape (*W*_*m *_= 12, *d *= 3). The insets are the first 100 steps (left) and the last 200 steps (right) of the optimization.

Can the fixed-*T *methods, that is, conventional Gibbs sampling with the temperature fixed to a lower value than 1, be a substitute for GibbsST? Certainly, temperature reduction of only 10% showed a considerable performance improvement in Fig. 3. However, lowering the temperature is not a universal solution because when the temperature is fixed to an exceedingly low value, sampling based on the temperature is rather similar to that of the inefficient steepest descent method. To demonstrate this vulnerability of fixed-*T *methods and the superiority of GibbsST, several fixed-*T *methods (*T *= 0.9, 0.8, 0.7, 0.6, and 0.5) are shown in comparison to the GibbsST algorithm in Fig. 4. A special dataset (*W*_*m *_= 12, *d *= 3) was prepared and used in this experiment because a dataset with a rough score landscape (only a slight difference exists between the global optimum and noise) is ideal for the current objective. In addition, a special temperature set (TLC 6, whose minimum temperature is as low as 0.46) is used to explore the possibility of lower temperatures. Fig. 4 shows the time-course of the average relative score (not the performance coefficient) of 100 datasets for various methods. The score is normalized with respect to the maximum score obtained for each dataset. The two insets show enlarged plots of the first 100 steps and the last 200 steps.

Lowering the temperature seems to be an ideal method to improve the convergence, as long as the score increase in the first 100 steps is concerned (left inset): the *T *= 0.5 setting shows a dramatically fast score increase in this region. However, the score increase of *T *= 0.5 eventually slows down: it is overtaken by *T *= 0.6 at ~200 steps, and by *T *= 0.7 at ~700 steps. In general, the greater the performance of a temperature setting is in the initial phase, the earlier the score ceases to improve. As a consequence, the scores of *T *= 0.5, *T *= 0.6 and *T *= 0.7 are stagnant in the final phase of optimization (right inset) and are perfectly in reverse order of their performance in the initial stage. The most probable reason for the fast score increase's subsequent performance deterioration is, of course, the local optima in the search space. Our proposal, GibbsST, is immune to such a general trend: its performance in the initial phase is not much poorer than that of best fixed-*T *methods, but its score in the final phase is better than any other setting tested. Considering that a small score difference may correspond to vastly different alignments in a rough score landscape of biological sequences, this level of difference in the final score is more than sufficient to clarify the superiority of GibbsST over fixed-*T *methods.

Although fixed-*T *methods do have simplicity and a limited usability as a substitute of GibbsST, a crucial problem exists in employing lowered and fixed temperature in Gibbs sampling. The temperature dependence of the behavior of the optimization process, like that shown in Fig. 4, is quite "nonlinear": there is no way to know the optimal temperature in advance. For that reason, even if a fixed-*T *setting better than GibbsST exists, the fixed-*T *setting is not likely to be available. The optimal temperature's possible dependence on characteristics of input sequences (discussed later) further complicates the situation, and increases the possibility of exceedingly lowered temperature. Consequently, the fixed-*T *method is very inconvenient as an acceleration method in pattern discovery problems. In addition to the fact GibbsST outperforms all sampling scheme tested in Fig. 4, it should be emphasized that GibbsST is the only method so far that has been proposed to utilize temperature lower than 0.9 without damaging the search robustness.

### Test on biological data

In this section, we demonstrate the usefulness of our algorithm for making more realistic TFBS predictions. Although our algorithm was quite effective for synthetic datasets, the statistical characteristics of natural promoter sequences may be very different from those assumed for synthetic datasets. Such a difference may demand further adjustment of the algorithmic parameters of simulated tempering (such as the temperature levels) according to the realistic potential landscape of natural promoters.

We selected six transcription factors of *Saccharomyces cerevisiae *for use in this test. There are two main reasons for this choice. First, very comprehensive information is available for this eukaryote from the Saccaromyces Cerevisiae Promoter Databases(SCPD) [21]. The promoter sequences, the regulatory relationships, and their evidence can be easily obtained from this curated database.

The second reason is related to the characterization of test data in terms of the global optimum. Using eight real TFBS of *Saccharomyces cerevisiae *and their flanking regions as examples, Friberg et al. [22] compared several different score-functions with respect to their sensitivity. In their test, the value of the score-functions were evaluated for all possible alignments in the flanking region and the rank of the biologically correct alignment (correct TFBS) was evaluated as an index of sensitivity of the score-functions. A scoring function called MAP (Maximum A posteriori Probability) yielded rank = 1 for five out of eight examples. Their definition of MAP was the one used in MDscan [11], which would be quite close to our current definition of score-function if it did not use the 3rd-order Markov model to describe the background sequences. Thus, now we have a list of transcription factors whose binding-sites have fairly large possibilities to be the global-optimum in terms of our current potential function.

**Figure 5:**
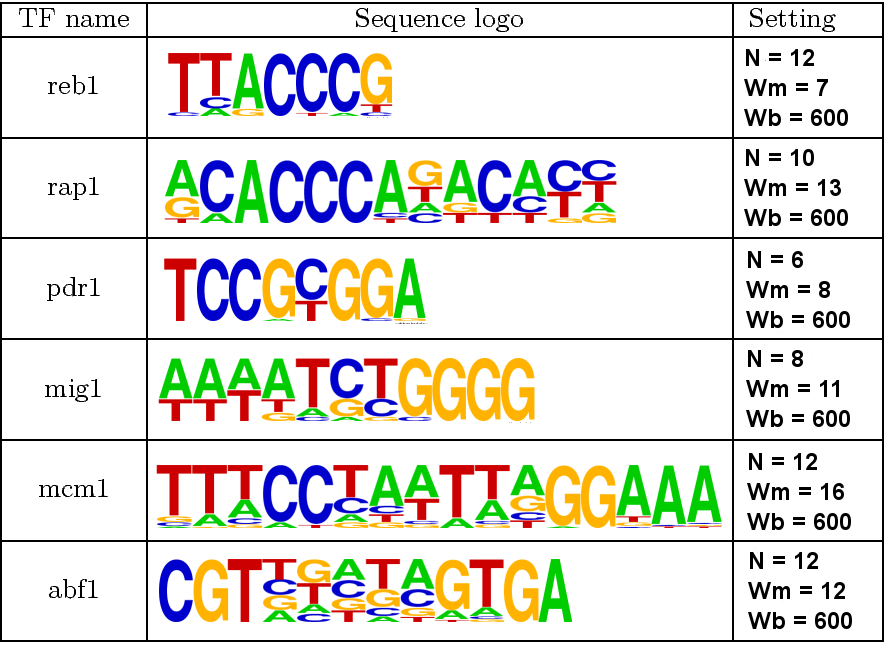
**Characteristics of biological datasets**. Characteristics of the datasets made from real *Saccharomyces cerevisiae *promoters: the names of the transcription factors, the sequence logos of their binding sites, and the parameters for the window selection.

The transcription factors we selected, reb1 [23], rap1 [24], pdr1 [25], mig1 [26], mcm1 [27], and abf1 [28], are introduced in Fig. 5. The other two examples were omitted because there were too few specific sites (gal4) and too few known binding sites (mac1) found in SCPD. For each transcription factor, 48 different datasets with different window placement were prepared. TFBS in minus strands were not excluded. According to Friberg et al. [22], the flanking regions of mcm1 and abf1 sites contain other sites associated with higher values of the current score function than the biologically correct binding sites. When the randomized placement of the window includes these non-target sites, the result may be an increased level of difficulty in the reconstruction of mcm1 and abf1 binding sites (see Method).

**Figure 6:**
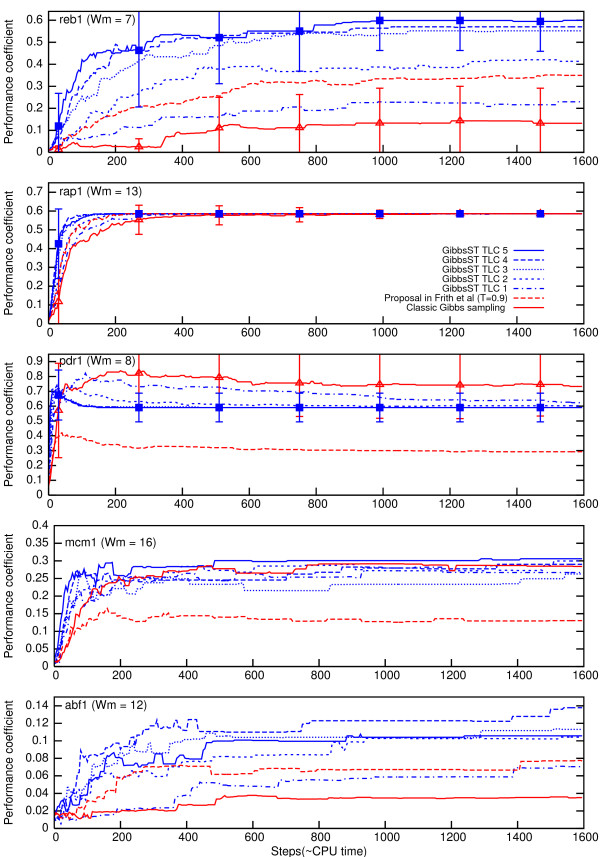
**Performance coefficient time course for biological datasets**. Average performance coefficient and its standard deviation (for classic Gibbs sampling and GibbsST with TLC5, except for mcm1 and abf1) for biological datasets.

The results are shown in Fig. 6 using the same format used for synthetic datasets. The lower average value of the performance coefficient can be attributed to binding sites of other transcription factors flanking the target TFBS, correlations in the background, and incompatibility between the score function and the target TFBS. In the cases of mcm1 and abf1, the average performance coefficient is especially low. The alignment snapshots of mcm1 were closely examined, and we found that the snapshots contain almost as many TTCC----GGAAA- and -TTTCC----GGAA as the biologically correct motif (TTTCC----GGAAA). These "phase-shifted-motifs" are considered to be a major form of local optima related to performance degradation [2]. When GibbsST was applied, both shifted-motif and correct-motif were sampled more frequently (that is how performance coefficient was increased), but their composition was not improved. It seems that GibbsST is not particularly suitable for solving the shifted-motif problem. The snapshots of pdr1 were also examined, but for this case, a totally different pattern of failure was identified (discussed later). GibbsST was unable to find any hit in the mig1 datasets, although this cannot be attributed to any defect of our algorithm, because, for these datasets, MEME also completely failed even when the correct *W*_*m *_was specified (by the "-w" option).

Still, the performance superiority of GibbsST over classic Gibbs sampling is clear in a majority of the tested cases. The general trend of a larger improvement for smaller *W*_*m *_and a larger variation among sites is not changed. Also, the best-performing temperature setting (TLC5) was generally unchanged from the case of the synthetic dataset. Although other settings performed best in some cases (rap1 and abf1), further consideration is required since some cases also showed a marked degradation of the overall performance. When *T *is fixed to 0.9, the results are classifiable into two categories. In the first category, which includes reb1, rap1, and abf1, the performance of *T *= 0.9 is identical to that in the synthetic datasets: the performance is better than *T *= 1.0 and worse than that of TLC2. In the second category, the result deviates surprisingly from that observed for synthetic datasets: the performance actually deteriorated when the temperature was lowered. For mcm1 and pdr1, encouraging the search algorithm to perform locally efficient sampling (by lowering the temperature) reduces the algorithm's efficiency in a global sense. A natural interpretation of this phenomenon is that the datasets of these two TFBS bear an especially complicated score landscape, which is confirmed later in Fig. 9. The optimal temperature setting seems to depend strongly on the characteristics of input sequences, and the adaptive nature of GibbsST might be an effective solution to alleviate the dependence.

#### pdr1

**Figure 7:**
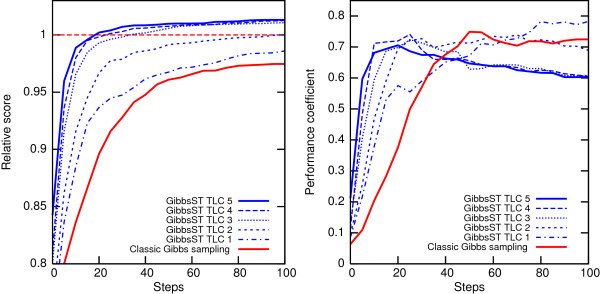
**Detail of the result for the transcription factor pdr1**. Time course of the value of score (left) and performance coefficient (right) obtained by GibbsST iteration for the dataset of transcription factor pdr1 (the first 100 steps).

It is worthwhile to take a close look at the result for pdr1, because it is quite different from the results for other transcription factors. The time courses of the relative score and performance coefficient in the first 100 steps are shown in the left and right halves of Fig. 7, respectively. The relative score is defined as the ratio of the current score to the score of the biologically correct answer. The plots show a quick increase of the performance coefficient and relative score followed by a quick convergence of the relative score (to ~1.014) and a sudden decrease of the performance coefficient for GibbsST (TLC3, 4 and 5) only. Slower and steadier convergences of the relative score (to ~1.002) and the performance coefficient were observed for classic Gibbs and GibbsST (TLC1 and 2). Apparently, GibbsST with appropriate temperature settings found a global optimum that was inaccessible to classic Gibbs sampling, although the global optimum was not biologically correct.

#### abf1

**Figure 8:**
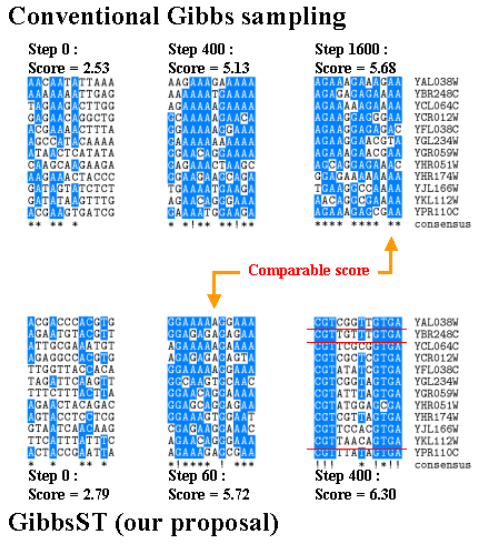
**Detail of the result for the transcription factor abf1**. Alignment snapshots for abf1 obtained using GibbsST and the classic Gibbs sampling algorithms. Note that the underlined segments have no biological evidence despite of their clear features of abf1 binding sites.

The result for abf1 is also interesting because of the low performance coefficient. In Fig. 8, alignments obtained for this case by classic Gibbs sampling and GibbsST are compared. These are snapshots taken from runs that yielded the highest scores. These examples show that GibbsST improves the quality of alignments far more efficiently than classic Gibbs sampling does. Here, GibbsST requires only 60 steps for the same level of progress, which requires 1600 steps of classic Gibbs sampling. In only 400 steps GibbsST achieved an alignment with clear features of the binding site of abf1 (CGT-----GTGA).

**Figure 9:**
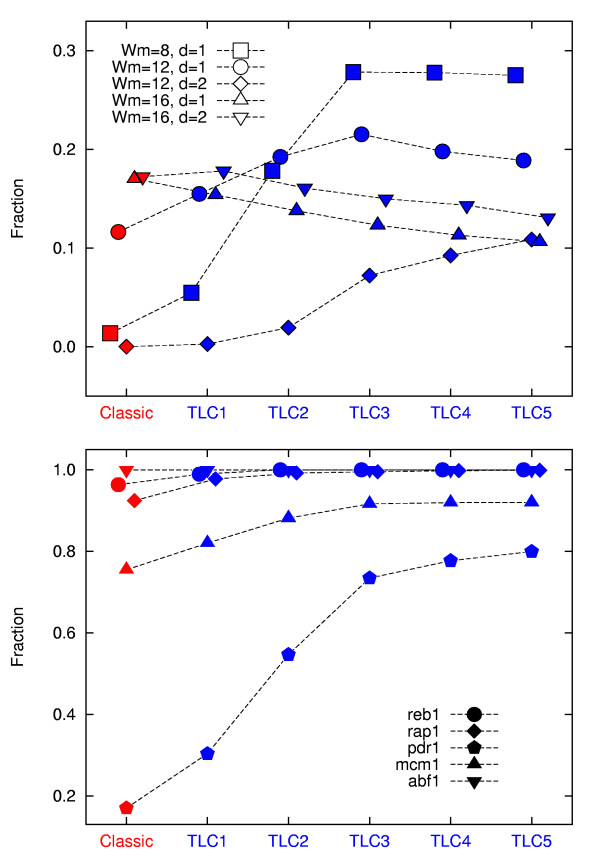
**Initial value dependence improved by GibbsST**. Fraction of initial values from which different algorithms and temperature settings were successful in our experiment.

The reason for the low performance coefficient is revealed by close examination of this alignment. The three segments marked in the alignment closely resemble the known abf1 sites, but they have no biological evidence in SCPD. These biologically non-confirmed sites engender the large disparity between the high score and low performance coefficient observed for abf1. Nevertheless, the high efficiency of GibbsST in convergence to a high-quality alignment is remarkable. We conclude that these data illustrate the strength of GibbsST in terms of the fast alignment improvement. They also show the limit of our current validation scheme in terms of the dependence on the "correct" answer.

### Temperature setting

The local optima dependence of optimization algorithms can also be analyzed as initial value dependence. An index of initial value dependence is the ratio of "successful" initial values to all initial values tested for a condition. This index is connected directly to the number of (random) initial values required (that is, roughly proportional to the CPU-time required) for finding one pattern in the solution space. We define the initial value as "successful" when a run started from an initial value reaches 99% of the score of the known global optimum (or biologically correct answer) at somewhere before 1600 steps.

The resultant index for synthetic TFBS is shown in the upper half of Fig. 9. Unlike the plots of the performance coefficient profile, these data show that the optimal temperature setting is not necessarily TLC5. It depends on the input sequence characteristics. For two conditions, TLC3 was optimal. For *W*_*m *_= 16, *d *= 2, TLC1 was optimal in terms of the local minima resistance. The "classic" algorithm was optimal for *W*_*m *_= 16, *d *= 1, but the difference between the "classic" algorithm and TLC1 was small. For these *W*_*m *_= 16 cases, the overall performance improvement of TLC3,4, and 5 shown in Fig. 3 derives mainly from the lower average temperature (quick convergence to the nearest local optimum), which is only a side effect of GibbsST. These cases illustrate the necessity for more sophisticated temperature settings, but GibbsST exhibits better overall performance than the classic method even for these cases, as shown by data of the performance coefficient.

As shown in the lower half of Fig. 9, TLC5 showed the greatest effect of alleviating the initial value dependence for biological test data. For reb1 and rap1, the situation tested was too easy to differentiate TLCs. For abf1, the data is not really reliable for the reason introduced in the previous section. It is noteworthy that the magnitude of enhancement of "successful" initial values was remarkably large for some conditions. For example, in the case of pdr1, GibbsST requires only one-fourth of the initial values of those required for classic Gibbs sampling (even greater enhancement was observed for the two synthetic cases). Our conclusion for temperature settings is as follows. The temperature setting TLC5 is the optimal selection when *W*_*m *_< 12 and large levels of pattern variation are expected. In such a case, a possibly lower minimum temperature than that of TLC5 should be considered for further performance improvement (as in TLC6). Temperature settings TLC3, TLC4 and TLC5 will work well for longer and rigid patterns (The precise best among these selections depends on input data). When *W*_*m *_≥ 16, TLC5 remains the best selection, but a better temperature setting should be devised for these cases regarding the initial value dependence. Alternatively, GibbsST should be tested for *W*_*m *_= 16 test data with larger pattern variations.

## Discussion

The performance of computational TBFS discovery can be enhanced by means of improvement of the search method in its own right. We assumed that a good search method must have resistance to local optima, to yield solution of better quality in fewer iterative steps. We also assumed that a good search method must not be strongly sensitive to the initial values. These goals were realized and demonstrated by our new algorithm, GibbsST. In the long run, this approach frees up computational resources for more biologically appropriate modeling of TFBS.

Many functions should be added to GibbsST. For example, non-OOPS occurrence models, better background models and automatic adjustment or scanning of *W*_*m *_are important. There is no fundamental difficulty in incorporating these functions into GibbsST. The standard method of estimation of the P-value [2,29] can also be implemented with ease, because the standard model and score definition is used in GibbsST.

Although we employed the relative entropy in the present work, there is a wide range of possible score functions to be combined with GibbsST. Because it is independent of the biological model, GibbsST only requires evaluation of

exp⁡−(U(new alignment)−U(old alignment))T     (7)
 MathType@MTEF@5@5@+=feaafiart1ev1aaatCvAUfKttLearuWrP9MDH5MBPbIqV92AaeXatLxBI9gBaebbnrfifHhDYfgasaacH8akY=wiFfYdH8Gipec8Eeeu0xXdbba9frFj0=OqFfea0dXdd9vqai=hGuQ8kuc9pgc9s8qqaq=dirpe0xb9q8qiLsFr0=vr0=vr0dc8meaabaqaciaacaGaaeqabaqabeGadaaakeaacyGGLbqzcqGG4baEcqGGWbaCdaWcaaqaaiabgkHiTiabcIcaOiabdwfavjabcIcaOiabb6gaUjabbwgaLjabbEha3jabbccaGiabbggaHjabbYgaSjabbMgaPjabbEgaNjabb6gaUjabb2gaTjabbwgaLjabb6gaUjabbsha0jabcMcaPiabgkHiTiabdwfavjabcIcaOiabb+gaVjabbYgaSjabbsgaKjabbccaGiabbggaHjabbYgaSjabbMgaPjabbEgaNjabb6gaUjabb2gaTjabbwgaLjabb6gaUjabbsha0jabcMcaPiabcMcaPaqaaiabdsfaubaacaWLjaGaaCzcamaabmaabaGaeG4naCdacaGLOaGaayzkaaaaaa@6184@

for its Gibbs sampling section, and the partition function, *Z*, for its temperature selection section. Any *U *is compatible with ST because evaluation of *U *is a totally encapsulated part of the algorithm. However, it should be noted that the concern about the computational complexity of the score function is reduced because of the substantial improvement of efficiency by ST. We can now employ score functions with more complex representation of biological specificity of binding sites. We are especially interested in rareness-based score functions [30], because of their improved biological sensitivity and relatively heavy computational burden.

Lower minimum temperatures and more sophisticated temperature scheduling should be tested, especially when GibbsST is applied to long rigid patterns. One trivial possibility that should be addressed is increasing *N*_*T*_, that is, the use of numerous small temperature steps. The problem with this simple idea is that temperature adjustments by means of small temperature steps would be unable to keep up with the rapid change of the alignment score. In fact, we frequently observed this phenomenon and the resulting severe degradation of performance during our preliminary testing of GibbsST. In other words, sudden and large changes in the value of the goal function are the most noteworthy features of TFBS discovery based on Gibbs sampling, when its combination with simulated tempering is considered.

This is only one example of the many possibilities of algorithmic design that should be explored before GibbsST is extended to other interesting problems of bioinformatics. We confined our study to the simplest of the tempering schemes and to elementary optimization of the temperature levels. Several improvements of the tempering scheme itself [31-33] are yet to be tested. However, we have secured a good starting point, TLC5, for exploration that is validated for both synthetic and biological promoter sequences. As evident in Figs. 3, 4, and 6, GibbsST is most effective for hidden patterns that have a high level of variation (*d*) compared to their length (*W*_*m*_) This fact is attributable to the shorter distance in the solution space between highly variable patterns and background noise compared to long and rigid patterns. This condition coincides with objectives of biological interest: sequence motifs with large variation. However, we were unable to validate GibbsST in a so-called "twilight-zone" of sequence pattern detection mainly because our test scheme depends on the success of MEME, although it is strongly anticipated that the performance gain in the twilight zone is even larger than that observed in the presented data. A better method of validation is necessary to advance our method in this direction. This direction should be advanced in combination with the better score function, evaluation of sensitivity, and specificity in an integrated manner.

Introduction of different methods into GibbsST is possible and promising. According to our preliminary test, the overall efficiency of GibbsST with the best temperature setting measured by the performance coefficient profile is roughly comparable to that of GibbsMotifSampler [8], a conventional Gibbs sampling method combined with a sophisticated selection of initial parameters (called "Near-optimum sampling"). Introduction of any successful initial alignment setting, not excluding the combinatorial approaches [34,35], into GibbsST as a preprocessing stage should be considered in the future as candidates for a very efficient pattern discovery program.

Seed-based initialization in search methods, that is, a preprocessing to find promising partial patterns, is quite useful to highlight the advantage of GibbsST. Even when not explicitly defined as such, all seed-based approaches assume that all partially correct solutions in the search space can be recognized and kept track of. In other words, a seed-based approach always assumes the availability of a complete catalog of all the deep basins illustrated in Fig. 1. Although nobody has ever reported any number statistics of basins in the concrete score landscape of the local-alignment problem, in some situations, such a catalog is going to be difficult to create. Such a breakdown of seed-based search methods is expected under two extreme conditions: when the score of the target pattern is too close to the noise-level, or the search space to be explored is simply immense. The first condition corresponds to the twilight zone. The second condition is mainly relevant to complicated models like patterns with special types of flexibility (e.g. variable length gaps). GibbsST can be extended and will be useful to any patterns in important subjects in bioinformatics (e.g. RNA and protein functional motifs) that meet either or both of these two conditions.

## Conclusion

Our new algorithm for TFBS discovery, GibbsST, is based on an adaptive adjustment of the search stringency and shows a much increased resistance to local optima. By combining Gibbs sampling and simulated tempering, GibbsST creates a robust platform for difficult pattern detection in biological sequences.

## Methods

### Algorithm details

Our current test code of GibbsST is implemented with the following algorithmic details. The row to be updated is selected in a round-robin fashion. The code internally prepares the minus strands of all input sequences such that the minus strands can be incorporated to the selection of new segment positions, if necessary. As mentioned above, the background model considers only the base composition (though the 2nd- or 3rd-order Markov model is fully compatible with ST), and the base composition is unchanged during the iteration. The temperature transition is carried out after each row update (other designs were tested, but all yielded poorer performances). The value of pseudocount in PWM is always fixed to 1.0 (a variable pseudocount is troublesome because it has a similar effect to variable *T*).

The value of *Z*_*i *_is numerically obtained by means of preliminary sampling. Each temperature level needs 4000 steps of preliminary sampling, which is enough to obtain equilibrium at each temperature. This preliminary Monte Carlo phase may be omitted in the future by, for example, a database of *Z*_*i *_for various conditions and interpolation formulae. Since Gibbs sampling is a type of Monte Carlo method, the pseudo random number generator is a crucial element. We selected a generator called the Mersenne twister [36], which is acclaimed for its fast generation and excellent randomness (very weak short-term order).

The number, length, and initial value selection method (random or Hamming distance based "seed") of independent runs can be changed by runtime options. Also, runtime options for temperature transition schemes are available, including the classic mode(*T *= 1.0, for a control). The current code is principally designed for investigation of local optima issues with many simplifications (e.g. *W*_*m *_must be specified by the user). Nevertheless, the code would be practical enough for realistic TFBS discovery if a proper combination of options is selected. A web server of this code will be available [37].

### Preparation of synthetic data

The synthetic "TFBS" sequences in our synthetic dataset were generated by adding *d *of random site mutations to a randomly generated consensus sequence of *W*_*m *_bp in length. N of such synthetic sequences were implanted into N of random background sequences each of which was *W*_*b *_bp long. The positions of the synthetic TFBS in these "windows", *y*_*i*_, were also random, and the direction was limited to the plus strand. When the value of *d *is large, the synthetic "TFBS" approaches to the twilight-zone of detection, and the implant score is comparable to that of the background noise; for large *d*, the implant is no longer guaranteed to be the global optimum. This is a large problem for the current experimental design because it means that the performance coefficient is no longer trustworthy.

To solve this problem, every synthetic promoter dataset was filtered by MEME 3.0.3 [29], which is a popular and reliable motif discovery tool. If the performance coefficient of MEME's answer was below a certain threshold (0.95), the dataset was abandoned. In the most difficult case, the acceptance rate of generated datasets was as low as 1%. Alternatively, increasing N could avoid this problem. However, this approach was not adopted because we wanted the N of synthetic dataset and biological datasets matched to each other.

### Preparation of biological data

The biological datasets were prepared as follows: beginning from the transcription initiation point, 1000 bp upstream regions were obtained from SCPD for each gene regulated by the target transcription factors. Removal of non-target TFBS and low-complexity sequences were not performed. The lists of correct binding sites were also obtained from SCPD (many of them are on minus strands). Minor manual editing was necessary on binding sites with length variation for conformity between the data and the current model (i.e. binding sites with variable length were removed). Then, a number of windows were randomly selected from these upstream regions such that each window contained at least one complete TFBS. The width of the windows (*W*_*b*_) was fixed to 600 bp, and the number of windows was adjusted such that an appropriate level of sensitivity was available.
